# Accumulation of inorganic polyphosphate enables stress endurance and catalytic vigour in *Pseudomonas putida* KT2440

**DOI:** 10.1186/1475-2859-12-50

**Published:** 2013-05-20

**Authors:** Pablo I Nikel, Max Chavarría, Esteban Martínez-García, Anne C Taylor, Víctor de Lorenzo

**Affiliations:** 1 Systems and Synthetic Biology Program, Centro Nacional de Biotecnología (CNB-CSIC), 28049 Madrid, Spain; 2 Escuela de Química, Universidad de Costa Rica, San José, 2060, Costa Rica; 3 Harvard College, Cambridge 02138, Massachusetts, USA

**Keywords:** Polyphosphate, *Pseudomonas putida*, Stress response, Energy homeostasis, Catalytic vigour

## Abstract

**Background:**

Accumulation of inorganic polyphosphate (polyP), a persistent trait throughout the whole Tree of Life, is claimed to play a fundamental role in enduring environmental insults in a large variety of microorganisms. The share of polyP in the tolerance of the soil bacterium *Pseudomonas putida* KT2440 to a suite of physicochemical stresses has been studied on the background of its capacity as a host of oxidative biotransformations.

**Results:**

Cells lacking polyphosphate kinase (Ppk), which expectedly presented a low intracellular polyP level, were more sensitive to a number of harsh external conditions such as ultraviolet irradiation, addition of β-lactam antibiotics and heavy metals (Cd^2+^ and Cu^2+^). Other phenotypes related to a high-energy phosphate load (e.g., swimming) were substantially weakened as well. Furthermore, the *ppk* mutant was consistently less tolerant to solvents and its survival in stationary phase was significantly affected. In contrast, the major metabolic routes were not significantly influenced by the loss of Ppk as diagnosed from respiration patterns of the mutant in phenotypic microarrays. However, the catalytic vigour of the mutant decreased to about 50% of that in the wild-type strain as estimated from the specific growth rate of cells carrying the catabolic TOL plasmid pWW0 for *m-*xylene biodegradation. The catalytic phenotype of the mutant was restored by over-expressing *ppk* in *trans*. Some of these deficits could be explained by the effect of the *ppk* mutation on the expression profile of the *rpoS* gene, the stationary phase sigma factor, which was revealed by the analysis of a P_*rpoS*_ → *rpoS*‘-’*lacZ* translational fusion. Still, every stress-related effect of lacking Ppk in *P. putida* was relatively moderate as compared to some of the conspicuous phenotypes reported for other bacteria.

**Conclusions:**

While polyP can be involved in a myriad of cellular functions, the polymer seems to play a relatively secondary role in the genetic and biochemical networks that ultimately enable *P. putida* to endure environmental stresses. Instead, the main value of polyP could be ensuring a reservoire of energy during prolonged starvation. This is perhaps one of the reasons for polyP persistence in live systems despite its apparent lack of essentiality.

## Introduction

*Pseudomonas putida* KT2440 is a non-pathogenic soil bacterium able to use a large number of C sources and colonize a wide variety of habitats. These characteristics reflect its metabolic diversity and the ability to adapt to many different physicochemical conditions. To cope with changing − and often harsh conditions, *P*. *putida* has developed a suite of molecular and physiological assets for counteracting environmental stresses. Yet, the mechanisms involved in such environmental robustness have been only partially elucidated [1,2]. Cataloguing them is thus important not just for understanding the abundance of *Pseudomonas* strains in sites afflicted by adverse environmental conditions, but also to take advantage of these features for biotechnological applications (e.g., biodegradation of xenobiotic compounds and/or biocatalysis through the expression of strong oxidative enzymes [3,4]). One extremely persistent component of all live systems which has been proposed to play a crucial role in the generic stress tolerance is inorganic polyphosphate (polyP) [5-8]. PolyP is a linear polymer composed by many tens or hundreds of inorganic orthophosphate (Pi) residues linked by high-energy phosphoanhydride bonds which is found through all the Tree of Life, thus accrediting a very ancient role in the shaping of live systems [7,9-11]. Although the precise physiological roles of polyP are not fully understood, the high-energy status of the phosphoanhydride bonds in this polymer has been related to a large number of relevant biological functions, e.g., [i] Pi reservoir [7], [ii] alternative ATP sink and/or source [7], [iii] chelator of divalent cations [12] and [iv] a key player in transcriptional regulation [7,13] in the stringent response [14,15] and many other cellular and metabolic processes [16-18] (including virulence [19,20]). But, which is the biological origin of the polymer and where and how does it map into the physiology of environmental bacteria like *P*. *putida*?

**Figure 1:**
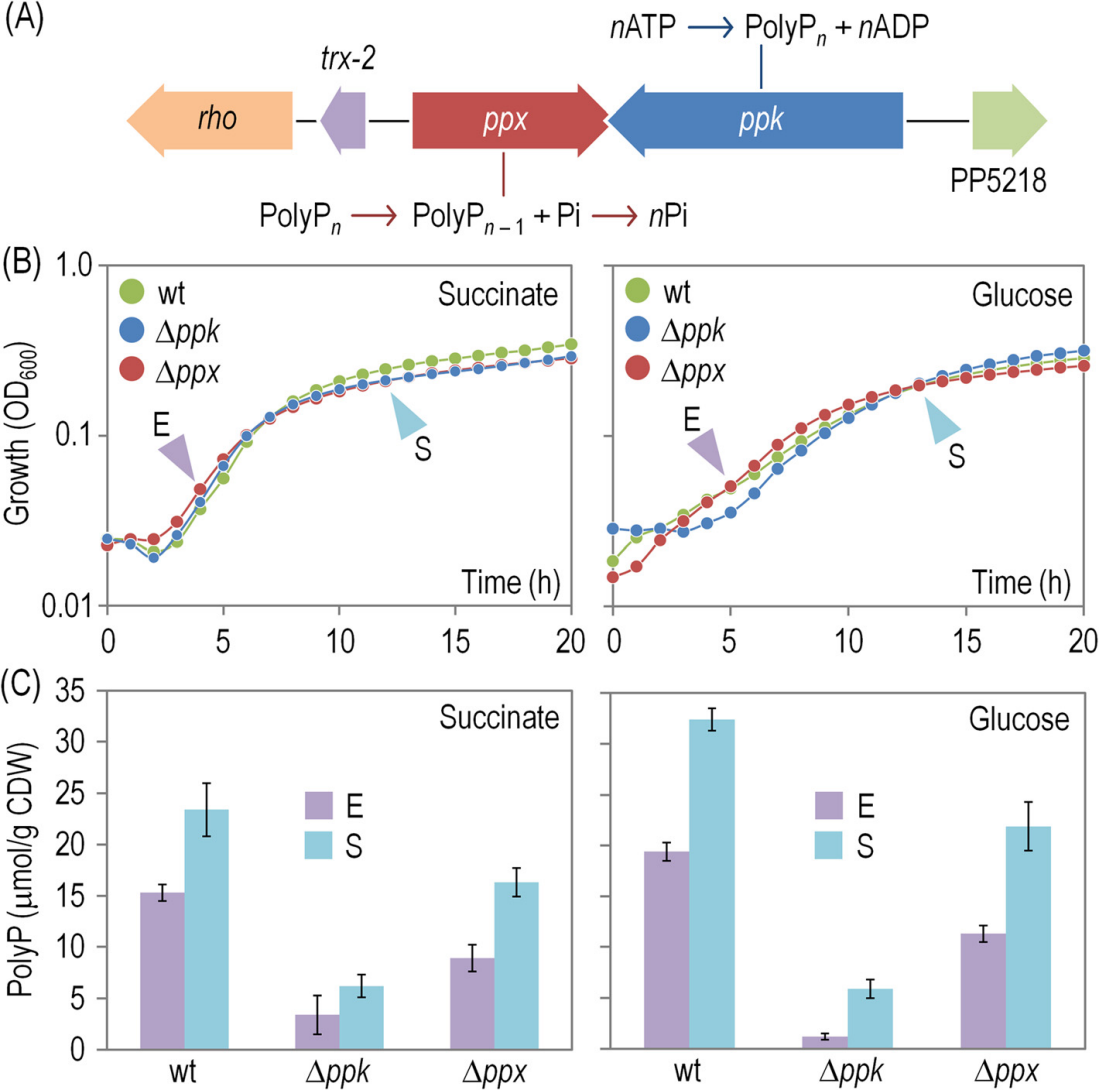
**Main bioreactions for polyphosphate (polyP) biosynthesis and degradation and accumulation levels in *P*. *putida *KT2440.** (**A**) Organization of genes involved in polyP metabolism in *P*. *putida*. Bioreactions catalyzed by polyP kinase (Ppk) and exopolyphosphatase (Ppx, annotated as a Ppx/GppA phosphatase) are outlined along with the gene encoding the corresponding enzyme involved in that transformation step. Relevant ORFs surrounding the polyP-genes locus are *rho* (encoding a Rho transcriptional terminator factor), *trx-2* (encoding a thioredoxin protein) and PP5218 (encoding a DedA family protein). Elements in this outline are not drawn to scale. (**B**) Growth curves for *P*. *putida* KT2440 (wt) and its Δ*ppk* and Δ*ppx* mutant derivatives developed under both glycolytic and gluconeogenic regimes. Experiments were conducted in M9 minimal medium with 0.2% (w/v) glucose or succinate as the sole C source as indicated in each plot. Time points in which the polyP content was assessed are identified with slanted arrows during both exponential growth (E) and in the stationary phase (S) (see below). Results represent the average of five independent replicates from at least three independent cultures, and error bars (consistently <10% of the means) are omitted here for the sake of clarity. (**C**) PolyP accumulation in *P*. *putida* KT2440 (wt) and its Δ*ppk* and Δ*ppx* mutant derivatives. Determinations were carried out in cells grown on M9 minimal medium with 0.2% (w/v) glucose or succinate as the sole C source as indicated in each plot either during exponential growth (E) and in the stationary phase (S). The polyP concentration was normalized to the cell dry weight (CDW) in each determination. Each bar represents the mean value of the polyP content ± SD of duplicate measurements from at least three independent experiments, and the asterisk identifies a significant difference at the *P* < 0.05 level (ANOVA).

Various pathways for the biosynthesis of polyP are known [7], the most widespread of them involving two families of polyP kinases termed Ppk (or Ppk1) and Ppk2. Ppk1 is encoded in the genomes of virtually all bacteria and it is responsible for the reversible polymerization of the γ-Pi residue of ATP molecules into the nascent polyP chain (Figure 1) [21,22]. In contrast, Ppk2 is supposed to preferentially catalyse the transformation of polyP back into various nucleosides triphosphate (NTPs, especially GTP) [23,24]. In addition to Ppk, there is another polyP-modifying enzyme: the so-called exopolyphosphatase (Ppx), that is in charge of polyP hydrolysis to Pi (Figure 1A). In any case, the basic reactions NTP + polyP_*n* – 1_ → polyP_*n*_ + *n*NDP and polyP_*n*_ ↔ *n*P_i_ are themselves wired to the cell physiology through a large number of enzymes that link the delivery of high-energy Pi moieties to various metabolic destinations, e.g., proteins and cofactors [25,26]. Yet, the enzyme that lies at the very top of any possible polyP functional network is Ppk1, conserved in virtually all prokaryotic species where the issue has been examined [7,11,27]. *Escherichia coli* cells lacking *ppk1* show deficiencies in long-term survival in the stationary phase of growth and lack resistance to oxidants, heat and osmotic challenges [17,19,20]. The same *ppk1* mutant of *P. aeruginosa* PAO1 lacked motility, was more sensitive to desiccation, more amenable to treatment with β-lactam antibiotics and performed worse forming biofilms than the wild-type counterpart [17,28,29]. Furthermore, polyP seems to be required for a full SOS response to DNA damage, and cells lacking this polymer fail to express stationary phase-induced stress genes [14,30]. Despite elusive mechanistic details, the emerging picture is that polyP seems to be involved in the tolerance to virtually all types of environmental stresses [14,30,31]. But whether this is a universal feature or only a peculiarity of the bacteria tested thus far remains uncertain.

In our quest to strengthen the stress tolerance of *P. putida* KT2440 as a microbial cell factory for biotechnological purposes [32-34], we have examined in depth the consequences of depleting the polyP pool in this bacterium. To this end, we constructed strains with altered polyP levels and evaluated a number of phenotypic traits that are relevant for both industrial and environmental biocatalysis. The data below show that a *ppk* knock-out mutant, in which the polyP content is very low, was generally more sensitive to virtually any type of environmental or metabolic stress than the wild-type strain. However, the same data bestows the NTP/polyP cycle a secondary – rather than primary role in the robust phenotypes that make *P*. *putida* so appealing as a host for whole-cell biocatalysis.

## Results and discussion

### The role of the *ppk-ppx* locus of *P*. *putida* KT2440 in the formation and degradation of polyP

As a first step to elucidate the physiological role of polyP in *P*. *putida* KT2440, we measured the intracellular levels of this polymer in cells grown on either glycolytic (i.e., glucose) or gluconeogenic (i.e., succinate) substrates as indicated in the Methods section. The intracellular polyP content was assessed *via* an assay method based on the metachromatic shift of a polyP/toluidine blue O complex [35]. Owing to the linearity of the polyP concentration against the ratio between the absorbance (*A*) measured at 530 nm and 630 nm (i.e., the *A*_530_/*A*_630_ ratio), polymer levels under different growth conditions could be directly compared. *P*. *putida* cells grown in M9 minimal medium produced polyP mostly during the exponential phase of growth with either C source (Figure 1C), but polyP sharply accumulated in the stationary phase, as would be expected for a reserve polymer. That the levels of polyP were similar on both glycolytic and gluconeogenic substrates suggest that its accumulation was more related to the intrinsic physiological state of the cells than to their metabolic regime. Furthermore, that a considerable amount of polyP was found in cells growing in a Pi-rich culture medium like M9 minimal medium (containing ~64 mM Pi [36,37]) pointed out that the buildup of the polymer had nothing to do with Pi availability or a possible dearth of Pi. In order to verify the origin of the thereby detected polyP formation, we inspected the genomic sequence of *P*. *putida* KT2440 [38,39] for gene(s) encoding Ppk. Ppk is the principal enzyme (if not the only one) that generates the polymer from the terminal Pi residue in ATP through a very conserved reaction (Figure 1A). The product termed PP5217 (ORF within coordinates 5,950,764-5,952,947 bp) is predicted to encode a 727 amino acid protein that shares 34% identity with Ppk from *E*. *coli*. The gene is adjacent to a converging ORF (within coordinates 5,949,275-5,950,777 bp) encoding a 500 amino acid-long protein (PP5216), with 39% identity to Ppx from *E*. *coli*, which runs an exothermic but otherwise futile reaction of polyP_*n*_ hydrolysis to polyP_*n* – 1_ + Pi and, eventually, to *n*Pi ([5], see also Figure 1). This genomic arrangement is conspicuously different from that of the same ORFs in enterobacteria, where *ppk* and *ppx* form a co-transcribed bicistronic operon [7]. The case of *P*. *putida* is instead comparable to that of *P*. *aeruginosa*, in which the same two genes are convergent and they partially overlap in their 3′-ends (Figure 1), suggesting that the transcriptional control of their expression is different from their enterobacterial counterparts. Although no other genes encoded in the *P*. *putida* genome qualified upfront for encoding the kinase and the phosphatase partners of the system (with the possible exception of PP0712), we verified their functionality by constructing strains deleted of each of these genes and measuring changes in the polyP contents of the mutant cells growing on glucose or succinate as explained above. Except for a significantly longer lag phase of the ∆*ppk* cells in the glucose-containing medium, the growth patterns of the mutant strains were practically indistinguishable from that of the wild-type strain (Figure 1B). However, the polyP level of the ∆*ppk* variant decreased drastically (70-90%) in all the growth conditions tested. This accredited the role of the encoded Ppk in the synthesis of the polymer but it also exposed that ≥20% of the whole contents of polyP of *P. putida* had a biochemical origin different from the reaction shown in Figure 1A (top). While this situation is similar to the state of affairs in other bacteria, the lowering in the polyP content brought about by the loss of *ppk* was considered high enough to trace any phenotypic consequences of the mutation to the lesser accumulation of intracellular polymer. Elimination of the polyP phosphatase gene (*ppx*) decreased – rather than increased the polymer levels as well, although to a much lesser extent (30-40%, depending on the C source used and the growth phase) (Figure 1C). This discarded the ∆*ppx* mutation as a strategy for artificially increasing polyP levels but it also suggested that the fate of much of the high-energy phosphate bonds of the polymer was not end-point hydrolysis (at least in the Pi-rich culture medium tested), but most likely delivery to other acceptors (e.g., proteins and/or small molecules) by means of specialized kinases [5]. Once the accumulation of polyP in *P. putida* was established and their perturbation by the ∆*ppk* and ∆*ppx* deletions quantified, we set out to examine the role of the polymer in a large collection of traits thay are key to a reliable performance in biotechnological settings.

### Gross phenotypic characteristics of a *P*. *putida* strain with low polyP content

**Figure 2:**
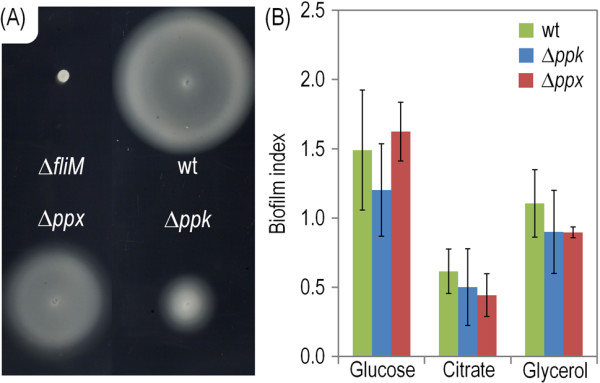
**Motility and biofilm formation by *P. putida *KT2440 and its Δ*ppk *and Δ*ppx *mutant derivatives.** (**A**) Swimming motility experiments performed in semi-solid M9 minimal medium agar plates for *P*. *putida* KT2440 (wt) and its Δ*ppk* and Δ*ppx* mutant derivatives. Two μl of a cell suspension of each strain (OD_600_ = 3.0) were inoculated onto the plate and photographed after 48 h of incubation at 30°C. A Δ*fliM* mutant (i.e., a flagellum-less strain) was used as a negative swimming control. (**B**) Biofilm formation. Assays were performed in multi-well plates using the crystal violet method explained in the Methods section. Comparison of biofilms formed by *P. putida* KT2440 (wt) and its Δ*ppk* and Δ*ppx* mutant derivatives was conducted by calculating the corresponding biofilm index values on each of the C sources tested (glucose, citrate and glycerol). Error bars represent the mean value of the biofilm index value ± SD of seven measurements from at least three independent experiments.

The physiological consequences of lacking Ppk and a low intracellular polymer were surveyed by passing the wild-type bacterium and its *ppk* variant through an abridged phenomic platform encompassing ~700 conditions covering a number of C, N and P sources along with a collection of stressful conditions, toxic compounds and antibiotics [40,41]. Table 1 shows the most informative phenotypes affected by the *ppk* deletion (for a complete dataset on phenotypic arrays, see in the Additional file 1: Figure S1). A number of such phenotypes were related to the use of some nucleosides monophosphate (NMPs) as the sole P source (e.g., 2′- and 3′-UMP, 3′- and 5′-AMP, Cys-*S*-P and 5′-TMP). Since polyP is a donor of high-energy phosphate for ATP synthesis under P-limiting conditions [5,42], the poor growth of the *ppk* mutant under these circumstances is likely to originate in its failure to synthesize ATP from NMPs. The key enzyme in this process (polyP:AMP-phosphotransferase [43]) could be the product encoded by PP1752, which is very similar to those orthologues found in other pseudomonads – an issue that deserves further studies. In any case, if the loss of polyP results in a lower load of high-energy Pi available *inter alia* for ATP synthesis, some phenotypes which depend directly on the load of this NTP are expected to be altered as well. One of the cell traits that consumes more intracellular ATP is swimming, as the motion of the flagelar motor puts away a considerable amount of molecules of this energy currency [29,44]. To verify the proposed link between polyP and a higher energy load we run swimming tests of the wild-type *P. putida* strain in semi-solid agar [0.3% (w/v)] plates along with its ∆*ppk* and ∆*ppx* derivatives. As anticipated, the cells deficient in polyP accumulation had a very limited flagellar activity, although they still had some motility when compared to a knock-out strain in the flagellar motor switch protein (FliM), which is entirely non-motile (Figure 2A). The plausible lowering of high-energy Pi available for rapid refilling of the ATP pool in ∆*ppk* cells advancing on the plate surface suffices to explain the slow-swim phenomenon. In contrast, the ∆*ppx* strain, although also flawed in motility, was far more proficient in the same test than the equivalent cells largely lacking the polymer. Since flagellar performance is also important for biofilm formation on abiotic surfaces [45], it came as little surprise that the ∆*ppk* strain colonized abiotic surfaces significantly worse than its wild-type counterpart in several growth conditions (Figure 2B). Although biofilm development is a complex trait that involves many factors beyond motility [46], our data suggest that polyP has an influence in the signal processing pathways that trigger surface attachment and maintenance of the biofilm structure.

**Table 1:** **Phenotypic microarray analysis of the *P*. *putida *Δ*ppk *mutant compared to the wild-type strain**

**Phenotype**^**a**^	**Positive/negative effect**
Thiophosphate	+
Potassium tellurite	+
Semicarbazide hydrochloride	+
2′-Uridine monophosphate (P)	−
3′-Uridine monophosphate (P)	−
3′-Adenosine monophosphate (P)	−
5′-Adenosine monophosphate (P)	−
Thymidine 5′-monophosphate (P)	−
Cysteamine-*S*-phosphate	−
Apramycin (Ab)	−
Ketoprofen (Ab)	−
Cefsulodin (Ab)	−
Azlocillin (Ab)	−
Oxacillin (Ab)	−
Nafcillin	−
3-Amino-1,2,4-triazole	−
Thallium (I) acetate	−
Cesium chloride	−

^a^*P*: phosphorus utilization; *Ab*: β-lactam antibiotic.

### PolyP-deficient *P*. *putida* cells are vulnerable to a suite of physicochemical stresses

**Figure 3:**
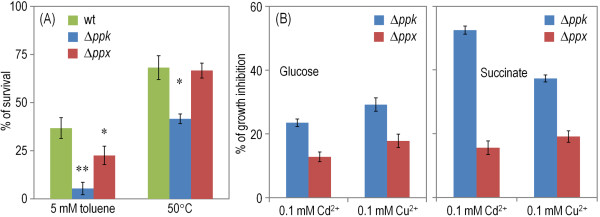
**Resistance to physico-chemical insults in *P*. *putida *KT2440 and its Δ*ppk *and Δ*ppx *mutant derivatives.** (**A**) Solvent tolerance and heat-shock response. Cells were cultured on LB medium and exposed to the different physicochemical stress agents as detailed in the Methods section. The percentage of survival was calculated as the fraction of colony-forming units after the treatment normalized to those in the untreated control experiment. Error bars represent the mean value of the percentage of survival ± SD of three independent replicates from at least two independent cultures, and asterisks identify significant differences at the *P* < 0.05 (*) or *P* < 0.01 (**) levels (ANOVA). (**B**) Growth inhibition by heavy metals in M9 minimal medium containing 0.2% (w/v) glucose or succinate as the sole C source. Experiments were performed in the presence of either 0.1 mM Cd^2+^ or 0.1 mM Cu^2+^ as indicated, and the percentages of growth inhibition were calculated at 24 h by comparing the growth of the mutants with that of *P*. *putida* KT2440. Error bars represent the mean value of the percentage of growth inhibition ± SD of three independent replicates from at least three independent cultures. All the differences in this parameter were significant (*P* < 0.05, ANOVA) as compared to the growth inhibition of *P*. *putida* KT2440.

Besides difficulties with the use of some P sources and the generation of molecules with high-energy phosphate, the phenomic analyses revealed an increased sensitivity of the ∆*ppk* mutant to specific types of stresses (in the Additional file 1: Figure S1). These included [i] exposure to cesium chloride and thalium, the toxicity of which is supposedly due to their similarity to potassium and interference with the synthesis of cysteine-containing proteins [47], [ii] the catalase inhibitor 3-amino-1,2,4-triazole and [iii] several β-lactam antibiotics, e.g., oxacillin, phenethicillin, nafcillin and azlocillin. The increased cidality exerted by these compounds suggested problems of the mutant in dealing with oxidative stress [30,48,49]. In contrast, other typical environmental hardships (i.e., alternative, not readily available C, N and S sources, high osmolarity and pH extremes) had no detectable effect on the ∆*ppk* strain. Three other types of stresses that are relevant under environmental or industrial conditions but are not included in the phenomic tests were examined separately, namely, exposure to aromatic solvents, tolerance to high temperatures (Figure 3A), and treatment with heavy metals in glucose- and in succinate-containing M9 minimal medium (Figures 3B and 3C). The growth assays added with Cu^2+^ and Cd^2+^ (Figures 3B and 3C) gave results entirely consistent with previous works reporting that polyP can act as a sink of divalent cations *in vivo*[12,50]. However, in any other respect the patterns of response of each of the strains to these insults were similar in most cases: the default tolerance level of the wild-type strain to metals, solvents or temperature decreased very significantly (although far from the point of collapse) in the ∆*ppk* strain, and to a lesser extent in the ∆*ppx* strain as well (Figure 3). While the effect of the stresses above on growing cells could be easily inferred from the corresponding normalized growth rates, we next wondered on whether the loss of polyP could also affect the survival and physiological state of bacteria after they had ceased to grow.

### PolyP is involved in extended survival in the stationary phase

**Figure 4:**
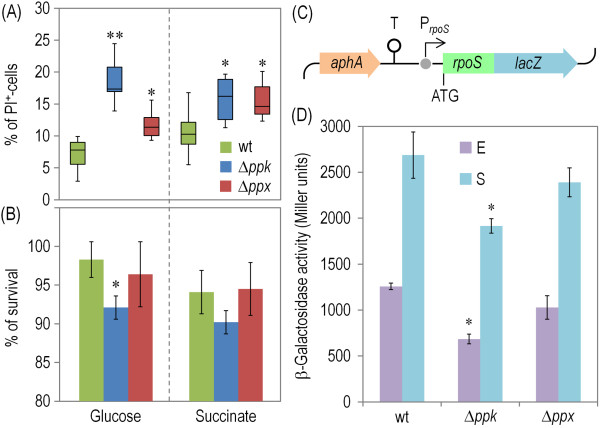
**Survival and P**_***rpoS***_**activity in *P*. *putida *KT2440 and its Δ*ppk *and Δ*ppx *mutant derivatives.** (**A**) Propidium iodide (PI) test to estimate cell viability in *P*. *putida* KT2440 (wt) and the Δ*ppk* and Δ*ppx* mutants. Stationary-phase cell suspensions grown on M9 minimal medium with either 0.2% (w/v) glucose or succinate were stained with PI and analysed by flow cytometry as detailed in the Methods section. Box plots represent the median value and the 1st and 3rd quartiles of the geometric mean values of quadruplicate determinations from three independent cultures, and asterisks identify significant differences at the *P* < 0.05 (*) or *P* < 0.01 (**) levels as assessed with the Mann–Whitney *U* test. (**B**) The ability of *P*. *putida* KT2440 (wt) and the Δ*ppk* and Δ*ppx* mutants to form colonies when transferred into fresh medium was evaluated by plating appropriate dilutions of the stationary-phase cell suspensions onto LB plates. Bars represent the mean value ± SD of three measurements from at least three independent experiments, and the asterisk identifies a significant difference at the *P* < 0.05 level (ANOVA). (**C**) Scheme of the relevant elements of the P_*rpoS*_ translational fusion borne by plasmid pMCH4. The T_0_ transcriptional terminator from phage λ is denoted as T. Elements in this outline are not drawn to scale. (**D**) Expression of the P_*rpoS*_ → *rpoS*‘-’*lacZ* translational fusion (β-galactosidase activity) monitored in *P*. *putida* KT2440 (wt) and the Δ*ppk* and Δ*ppx* mutants. Cells were grown on M9 minimal medium with 0.2% (w/v) glucose and 150 μg/ml kanamycin, and harvested during exponential growth (E) or in the stationary phase (S). Bars represent the mean value of the reporter activity ± SD of duplicate measurements from at least three independent experiments. Asterisks identify significant differences at the *P* < 0.05 level (ANOVA).

One of the prevailing hypothesis to explain the prevalence of polyP through all the phyla of Life is that the polymer acts as an energy reservoir once other cellular supplies have been depleted [7,9,20]. Such a situation would help maintaining a dormant − but still viable physiological state for a prolonged period of time [5,9,51]. To explore this scenario in *P*. *putida*, we inspected the physiological state of individual cells in cultures that have well entered into the stationary phase [i.e., 24 h after having reached their maximum optical density at 600 nm (OD_600_) values] in M9 minimal medium amended with either glucose or succinate. We resorted to the viability test with propidium iodide (PI), based on dye exclusion, as explained in the Methods section. Cells endowed with intact, polarized membranes are able to interact and exclude charged molecules like PI, while dead or seriously damaged bacteria become stained with the dye (which is a strong DNA intercalating agent) [52]. A significant share of the population of stationary wild-type cells (~10-15%) were stained with PI at the time of sampling (Figure 4A). This relatively high proportion of dead cells partially originates in the stochastic induction of the prophages borne by the *P. putida* genome [38] at late growth stages (Martínez-García *et al*., in preparation). In any case, these figures of PI-positive *versus* PI-negative bacteria provided a reference on how to compare the same trait in ∆*ppk* cells. The loss of Ppk resulted in a significant increase in the number of PI-stained cells (Figure 4A), meaning more mortality in stationary phase. The effect of *ppk* loss was slightly more pronounced in cells pre-grown on glucose, perhaps because intrinsic differences in the physiological state of the cells as compared to those grown on succinate [53]. Consistently with the data above, the absence of exopolyphosphatase (i.e., in the ∆*ppx* mutant) had little effect on the phenotypes under examination. When the same cultures were plated for measuring the actual viable counts (Figure 4B), the PI-staining data of Figure 4A was faithfully reflected in the number of viable colonies. Such a match between the PI-staining data and the viable count figures argued against a significant role of polyP in the possible emergence of persistent cells or other types of viable-but-not-culturable regime in *P*. *putida.* Yet, the interplay between the polymer and the persistence phenomenon, which has been suggested for many other bacteria [5,7,9,20,51], still warrants some clarification. In order to ascertain whether the observed differences in stationary phase survival could be traced to changes in polyP accumulation we run a complementation test. For this, we constructed plasmid pSEM-*ppk*, in which the *ppk* gene of *P*. *putida* KT2440 is expressed under the control of a promoter inducible with 3-methylbenzoate (see Methods for details). The wild-type strain carrying the empty vector pSEVA238 [54] and the Δ*ppk* variant with either pSEVA238 or pSEM-*ppk* were grown as described above, *ppk* induced and the percentages of PI-stained cells compared. As expected, the PI-positive populations in cultures of *P*. *putida* KT2440/pSEVA238 and its Δ*ppk* counterpart carrying pSEM-*ppk* were similar, the values comparable to those of Figure 4A for the wild-type strain. Consistently, the share of PI-positive cells of the complemented strain was significantly lower than the Δ*ppk* sample with the empty vector (not shown).

The results above establish an unequivocal link between polyP accumulation and cell viability in the stationary phase, but they say nothing on the possible mechanism(s). Since many of the stationary-phase phenotypes observed in *Pseudomonas*[17,28,29] and many other Gram-negative eubacteria [19,20,51,55,56] largely rely on the action of the alternative σ^S^ factor (also termed RpoS or σ^38^), we wondered whether a low content of polyP could affect expression of *rpoS* – as previously observed in *E*. *coli*[14,30]. To test this hypothesis, we constructed a translational fusion between the 87 leading structural codons of *rpoS* and a truncated *lacZ* (i.e., *rpoS*‘-’*lacZ*) preceded by a 774-bp genomic DNA fragment upstream of the first ATG, which we introduced in order to capture any possible *rpoS* regulatory element, whether transcriptional or translational [57]. The resulting fusion (P_*rpoS*_ → *rpoS*‘-’*lacZ*, Figure 4C) was assembled in the low-copy-number plasmid termed pMCH4 (see Methods for details), passed to the wild-type *P. putida* strain and its ∆*ppk* and ∆*ppx* derivatives, and the levels of β-galactosidase measured in exponential and stationary cultures in M9 medium with glucose as C source. β-Galactosidase assay results revealed a significant reduction (~40-50%) of P_*rpoS*_ activity in the Δ*ppk* mutant and an expectedly less intense effect in the Δ*ppx* strain (Figure 4D). These results not only suggested that polyP plays a role in the intricate signal transduction pathway that leads to optimal expression of the stationary σ factor, but also that the loss of viability in non-growing cells (see Figures 4A and 4B) could be traced to the reduced levels of RpoS that are inferred from the activity of the P_*rpoS*_ → *rpoS*‘-’*lacZ* fusion in ∆*ppk* cells.

### PolyP restrains DNA damage

**Figure 5:**
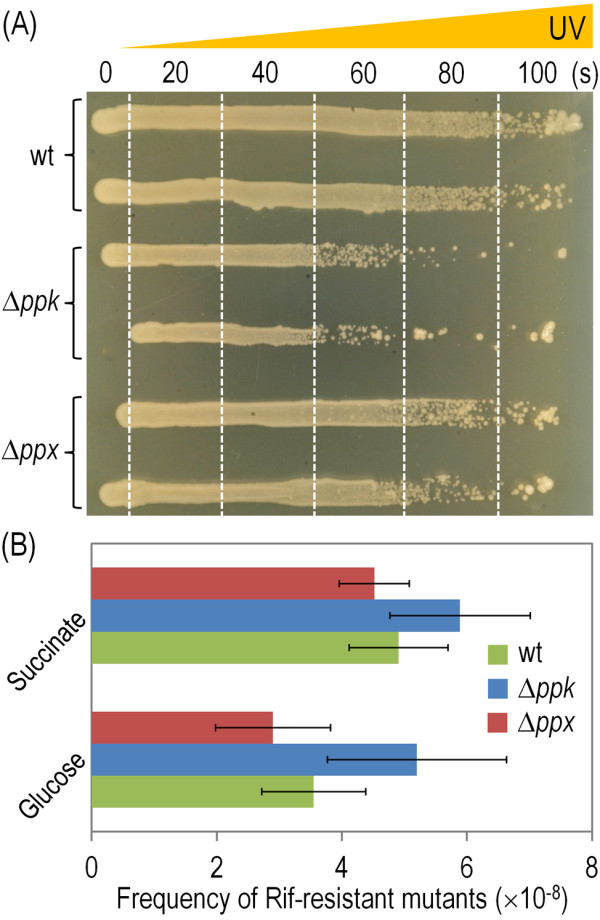
**UV-light survival and mutagenesis in *P*. *putida *KT2440 and its Δ*ppk *and Δ*ppx *mutant derivatives.** (**A**) UV sensitivity tests were performed in *P*. *putida* KT2440 (wt) and its Δ*ppk* and Δ*ppx* mutant derivatives streaked out onto LB plates by means of a gradient of UV irradiation (with exposure time ranging from 0 to 100 s). Irradiations were performed at 254 nm (400 μW/cm^2^), and the boundaries of different irradiation periods of time are indicated by vertical dashed lines. (**B**) Frequency of spontaneous Rif-resistant mutants in stationary-phase cells of *P*. *putida* KT2440 (wt) and its Δ*ppk* and Δ*ppx* mutant derivatives grown on M9 minimal medium with either 0.2% (w/v) glucose or succinate. Frequencies were calculated by dividing the average number of Rif-resistant colonies by the total number of viable cells in the same culture. Bars represent the mean value of the frequency of Rif-resistant clones ± SD of duplicate measurements from at least two independent experiments.

DNA damage is one of the most common environmental insults experienced by bacteria either in the environment or in a biocatalysis setting. The injury might be direct [e.g., ultraviolet (UV) radiation from sunlight and/or exposure of DNA to intercalating or reactive agents] or indirect (e.g., brought about by side-production of reactive oxygen species during redox catalysis [58,59]). Since the various activities of the RecA protein require ATP [60-63], the functioning of the SOS response is a process that consumes considerable amounts of this energy currency. It is therefore anticipated that cells lacking *ppk* and thus unable to retrieve quickly high-energy Pi for ATP regeneration will do worse when facing DNA damaging conditions than the wild-type strain. The simple test of Figure 5A pictures such a circumstance: the *P*. *putida* ∆*ppk* mutant was visibly more sensitive to direct UV irradiation than the wild-type strain, while the ∆*ppx* mutant was only minimally affected. These results were roughly consistent with the emergence of rifampicin (Rif) resistant clones in the various strains, a standard test for measuring mutagenesis rates under various conditions [64]. The ∆*ppk* cells seemed more prone to mutate to Rif-resistance than the other counterparts (Figure 5B), the phenomenon being more evident on glucose-grown cells. Although the interplay between DNA damage, SOS response, polyP accumulation and mutagenesis (as exposed through the emergence of Rif-resistant derivatives) has been explained in other bacteria on the basis of multiple mechanisms [7,30,42], the lowering of high-energy Pi traffic between the polymer and ATP could account for the entirety of the UV-sensitivity and high mutagenesis phenomena.

### Catalytic vigour of *P*. *putida* cells with altered levels of polyP accumulation

**Figure 6:**
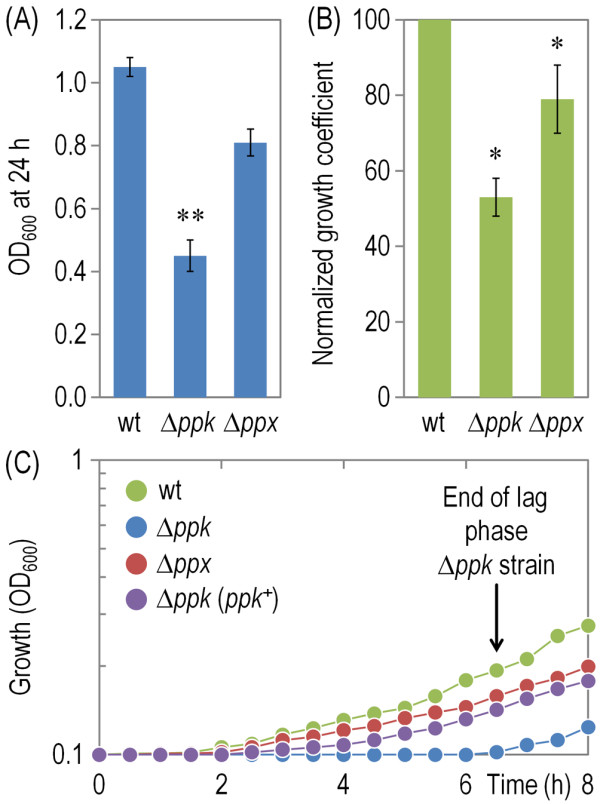
**Catalytic vigour test for *P*. *putida *KT2440 and its Δ*ppk *and Δ*ppx *mutant derivatives.** (**A**) Final biomass concentration (estimated from OD_600_ readings) for cultures of *P*. *putida* KT2440 (wt) and its Δ*ppk* and Δ*ppx* mutant derivatives grown in M9 minimal medium containing *m*-xylene as the sole C source. Bars represent the mean value of the OD_600_ readings ± SD of duplicate measurements from at least three independent experiments, and asterisks (**) identify significant differences at the *P* < 0.01 level (ANOVA). (**B**) Normalized growth coefficients for cultures of *P*. *putida* KT2440 (wt) and its Δ*ppk* and Δ*ppx* mutant derivatives grown in M9 minimal medium containing *m*-xylene as the sole C source, representing the fraction of the specific growth rate attained by mutant cells when compared to that computed for *P*. *putida* KT2440 (arbitrarily set to 100%). Bars represent the mean value of the normalized growth coefficient ± SD of triplicate measurements from at least four independent experiments, and asterisks (*) identify significant differences at the *P* < 0.05 level (ANOVA). (**C**) Growth kinetics for cultures of *P*. *putida* KT2440 (wt) and its Δ*ppk* and Δ*ppx* mutant derivatives, as well as the Δ*ppk* mutant complemented with *ppk* in *trans* (*ppk*^+^), grown in M9 minimal medium containing *m*-xylene as the sole C source. Expression of *ppk* (under control of a XylS/*Pm* element in plasmid pSEM-*ppk*) was induced by addition of 2.5 mM 3-methylbenzoate to the cultures upon inoculation. Growth trajectories in control experiments, run with the same strains carrying pSEVA238 [54], the empty expression vector used to complement *ppk*, were indistinguishable to those shown in this figure (not shown). Results represent the average of three independent replicates from at least two independent cultures. Error bars (<20% of the means) were omitted for the sake of clarity.

*P*. *putida* KT2440 is the original host of the pWW0 TOL plasmid for complete biodegradation of toluene and *m-*xylene into intermediates of the central metabolism [65,66]. This metabolic trait epitomizes every feature that is desirable in a whole-cell bacterial biocatalyst: high solvent tolerance, robust performance of oxidative biochemical reactions involving a number of cofactor-consuming redox transformations and full compatibility with the indigenous physiology. The growth of *P*. *putida* carrying the TOL plasmid on *m*-xylene as the sole C and energy source is thus a merged descriptor of the catalytic vigour of the strain and a reference to quantify the contribution of specific cellular devices to the optimal outcome. We employed such a test for measuring the total effect of low polyP levels in such a catalytic vitality (Figure 6). The results yield three important parameters in this respect. First, the OD_600_ values at 24 h reflect the overall conversion of substrate to biomass in each of the strains tested under the period of time under examination. The substrate-to-biomass conversion was affected in the ∆*ppk* mutant, while the ∆*ppx* strain was not much upset under the same growth conditions. These figures roughly match the normalized growth coefficients of the same strains (Figure 6B), and could be interpreted as if much of the metabolic currency generated by the biodegradative pathways [e.g., ATP, NAD(P)H and metabolic building blocks] were spared in enduring stress rather than being funnelled into biomass. Finally, the first stage of the growth curves for the three strains (Figure 6C) reveals a conspicuously long lag phase before the cells had any chance to take growth off. This extended period of time in ∆*ppk* cells before entering exponential – if still slow growth was observed also in glucose-grown cells (Figure 1B), was reported in other bacteria as well. Although this phenomenon might have many explanations, it has been attributed to a connection between polyP and ppGpp [14,67,68] that would make cells impaired in the *de novo* synthesis of amino acids [5,69]. We also followed the growth of the Δ*ppk* mutant strain complemented pSEM-*ppk* (Figure 6C). Expression of *ppk* in *trans* restored the growth of the strain to levels similar to those observed in the wild-type bacteria, with a significant reduction of the pronounced lag phase observed in the mutant carrying the empty vector. Our data above exposes a definite role of polyP in maintenance of the physiological robustness needed for running the multi-tiered biotransformations encoded by the TOL plasmid.

## Conclusion

The loss of the *ppk* gene that, as in many other organisms, lowers the polyP content of *P*. *putida,* is translated into a general phenotype of vulnerability to a large number of stresses. However, although such a weakness to face harsh physicochemical conditions makes ∆*ppk* cells unfit for hosting oxidative reactions or for facing environmental insults, in no case we found circumstances where a low polyP level was directly translated into cell death. These observations do not ultimately clarify whether the polymer is essential for viability, mainly because ∆*ppk* cells still contain a significant level (~15%) of polyP as compared to the wild-type strain. It is thus certain that polyP can be produced, albeit to a much lower cellular content, by other routes than by straight addition of high-energy Pi from ATP. PolyP has been reported to deliver such Pi not only to NMPs and NDPs [5,7] but also to NAD^+^[5,70], sugars [71-73] and proteins [5,43]. In addition, there seems to be a connection between polyP, the ppGpp alarmone and the stationary σ factor RpoS [14,56,74-76]. Measurement of P_*rpoS*_ activity using a *lacZ* translational fusion indicated a significant downregulation of the gene in the ∆*ppk* background (Figure 4D) confirming a certain role for polyP in RpoS induction/functioning. The increase in stationary phase mortality could be explained under such terms, although the effects were far less pronounced than those reported for bacteria as close to *P*. *putida* as *P*. *aeruginosa*. But the interplay between polyP and metabolic stress might still be more intricate. The Ppx protein is highly homologous to GppA, an enzyme with exopolyphosphatase and 5′-phosphohydrolase activity on the alarmone (p)ppGpp [6,25,42]. This plausible connection may account for the lack of conspicuous phenotypes in ∆*ppx P*. *putida*, as the effects of lacking the phosphatase might be buffered by other cellular activities. Tracing polyP metabolism to the activity of Ppk and Ppx only seems thus being an oversimplification.

One way or the other, that *P*. *putida* strains with altered polyP levels could still cope with the drastic drain of a major resource for stress endurance and stationary phase survival adds to the notion that this species is endowed with an extraordinary robustness that makes it an appealing choice as a host of biotransformations in both industrial and environmental biotechnology.

## Methods

### Bacterial strains, culture media and growth conditions

All the strains used in this work were derived from wild-type strain *P*. *putida* KT2440. Bacteria were grown aerobically in either LB medium or M9 minimal medium [36,37] added with 0.2% (w/v) of either glucose, succinate, citrate or glycerol as the sole C and energy source (as specified in the text) at 30°C and shaking at 170 rpm. Unless otherwise stated, cultures were developed in 250-ml Erlenmeyer flasks containing 50 ml of the corresponding culture medium. Inocula were prepared by growing cells for 18 h in the corresponding culture medium as explained above. Cultures were inoculated at an starting OD_600_ ~0.05 and growth was periodically monitored by following the OD_600_ of culture aliquots diluted as appropriate. Whenever needed, 3-methylbenzoate was added at 2.5 mM to culture media to induce the *Pm*-driven expression of *ppk* (see below).

A kanamycin-resistant derivative of the pWW0 TOL plasmid, originally termed pWW0-161::Tn*5*-*68* (herein referred to as pWW0-Km), has been described previously by Franklin *et al*. [65]. In some experiments, plasmid pWW0-Km was transferred into *P*. *putida* strains by triparental mating [77]. Kanamycin-resistant transconjugants were selected for their ability to grow on M9 minimal medium agar plates containing 5 mM sodium 3-methylbenzoate as the sole C source (details not shown). Overnight cultures of the strains carrying pWW0-Km were developed in M9 minimal medium containing succinate and *m*-xylene. Cells from these cultures were concentrated by centrifugation (4,000 rpm, 10 min, room temperature) and resuspension in M9 minimal medium, and the cell suspension used to inoculate 15 ml of fresh M9 minimal medium saturated with *m*-xylene as the sole C source (starting OD_600_ ~0.1). In these experiments we used 150-ml Erlenmeyer flasks equipped with teflon screw caps to prevent *m*-xylene loss by evaporation. Growth of the strains at 30°C and 170 rpm was followed for 24 h, and growth parameters were calculated as explained elsewhere [53]. In the complementation studies, control strains were transformed with the corresponding empty vector (see text for details).

For phenomic tests of the *P*. *putida* Δ*ppk* strain we resorted to the Phenotype MicroArray™ (PM) technology [40,41]. An abridged PM profiling was carried out by Biolog Inc. (Hayward, CA, USA) using a comprehensive set of 20 plates (see Additional file 1: Figure S1 for details) designed to reveal metabolic and stress-endurance differences between the wild-type *P. putida* strain and its Δ*ppk* derivative with respect to respiration (i.e., tetrazolium dye reduction) under a variety of growth conditions and sensitivities to several compounds.

### Construction of Δ*ppk* and Δ*ppx* deletion strains and cloning of *ppk* from *P*. *putida* KT2440 for complementation studies

Knock-out mutants were constructed using the allelic replacement method developed by Martínez-García and de Lorenzo [78]. Appropriate oligonucleotides (Additional file 1: Table S1) were used to amplify 400-bp flanking regions around *ppk* (Δ*ppk*-TS1-F and Δ*ppk*-TS1-R, and Δ*ppk*-TS2-F and Δ*ppk*-TS2-R) and *ppx* (Δ*ppx*-TS1-F and Δ*ppx*-TS1-R, and Δ*ppx*-TS2-F and Δ*ppx*-TS2-R). The approximately 800-bp amplification products obtained with the external oligonucleotides were first cloned into vector pEMG [78], giving rise to plasmids pEMGΔ*ppk* and pEMGΔ*ppx*. These suicide vectors were isolated and individually mated into *P*. *putida* KT2440 (carrying the helper plasmid pSW-I [79]). Cointegrate clones were grown overnight in 5 ml of LB medium containing 500 μg/ml ampicillin and 15 mM sodium 3-methylbenzoate (as an inducer of the I-*Sce*I − mediated recombination) and plated onto LB agar medium. Isolated colonies were re-streaked onto either LB agar or the same medium containing 50 μg/ml kanamycin to check for the loss of cointegrated plasmids. Kanamycin-sensitive clones were analyzed by colony PCR to identify clones in which either *ppk* or *ppx* had been deleted (details not shown). Finally, plasmid pSW-I was eliminated after three consecutive passes in LB medium. Elimination of the helper plasmid was verified in all cases by direct colony PCR amplification using the oligonucleotides pair pSW-F and pSW-R (Additional file 1: Table S1). For complementation assays, the entire *ppk* coding sequence (2,184 bp) was amplified with oligonucleotides *ppk*-F and *ppk*-R (Additional file 1: Table S1). The resulting amplicon was digested, ligated to the polylinker of the expression plasmid pSEVA238 [54] as an *Eco*RI-*Bam*HI fragment (giving rise to pSEM-*ppk*) and subsequently transformed and propagated in *E. coli* DH5α [37]. In the resulting plasmid, expression of *ppk* is driven by the XylS/*Pm* system. Plasmid pSEM-*ppk* was isolated from *E*. *coli* and electroporated in *P. putida* KT2440 and its Δ*ppk* mutant derivative as described elsewhere [78,80].

### Determination of polyP

PolyP was extracted from the cells by a modification of the protocol described by Ault-Riché *et al*. [35]. Appropriate aliquots of bacterial cultures were spun down and the biomass was resuspended in 500 μl of lysis buffer (4 M guanidine thiocyanate · HCl in 0.5 M Tris · HCl [pH 7.5]) prewarmed at 95°C. The suspension was heated at 95°C for 5 min, after which 30 μl of 10% (w/v) sodium dodecyl sulfate, 500 μl of 95% (v/v) ethanol, and 10 μl of Glassmilk (MP Biomedicals LLC, Illkirch, France; cat. # 1001–404) were sequentially added with vigourous vortexing. Samples were centrifuged in a benchtop centrifuge (12,500 rpm, room temperature, 30 s), supernatants were discarded, and the polyP-containing silica was washed three times with 500 μl of wash buffer (5 mM Tris · HCl [pH 7.5], 50 mM NaCl, 5 mM EDTA, and 50% (v/v) ethanol). Pellets were suspended in 100 μl of nucleic acids lysis buffer (1 mg/ml each of DNase I and RNase A in 50 mM Tris · HCl [pH 7.5]) and incubated at 37°C for 30 min. Silica was again pelleted and suspended in 300 μl of a 1:1 (v/v) mixture of lysis buffer and 95% (v/v) ethanol. After a 5-min incubation at room temperature, silica was recovered and treated twice with 500 μl of wash buffer. PolyP was eluted from the silica by suspending the pellets in 50 μl of 50 mM Tris · HCl (pH 8.8), incubating the suspensions at 95°C for 5 min, and saving the supernatant after centrifuging the samples as described above. This procedure was repeated two times, and the three 50-μl eluates were finally pooled together in a clean tube. PolyP was quantified spectrophotometrically by measuring the metachromatic shift of toluidine blue O from 630 to 530 nm. A 100-μl aliquot of the polyP-containing eluates, obtained as explained above and diluted when necessary, were added to 900 μl of a freshly-prepared dye solution consisting of 6 mg/ml toluidine blue O in 40 mM CH_3_COOH. Calibration curves spanning the range 4–64 nmol of polyP per assay were routinely run using a commercial polyP standard with an average chain length of 45 phosphate residues (Sigma-Aldrich Co., St. Louis, MO, USA; cat. # S4379). PolyP length in the assay was calibrated using polyP_14_ (short-chain polyP), polyP_60_ (medium-chain polyP) and polyP_130_ (long-chain polyP) authentic standards from RegeneTiss Inc., Okaya, Japan (kindly provided by Toshikazu Shiba). After addition to the dye solution, samples were incubated at room temperature for 15 min. The ratio *A*_530_/*A*_630_ was calculated by assessing the corresponding *A* values in each sample. PolyP levels in the samples were obtained by direct interpolation of the corresponding *A*_530_/*A*_630_ ratio into the calibration curve, and expressed as nmol polyP per g cell dry weight (CDW).

### Metal sensitivity tests

Cells were grown as described above and used to inoculate fresh M9 minimal medium containing either glucose or succinate, and amended with either 0.1 mM of CdCl_2_ or CuCl_2_ (added from concentrated aqueous solutions pre-sterilized by microfiltration). Cultures were distributed in 200-μl aliquots in 96-well plates (Nunclon™Δ Surface; Nunc A/S, Roskilde, Denmark). Bacterial growth was periodically estimated by monitoring the OD_600_ in a SpectraMax Plus384 Microplate Reader (Molecular Devices, Sunnyvale, CA). Plates were incubated at 30°C for 24 h with 1 min of orbital shaking every 30 min, just prior to each OD_600_ measurement. The percentage of growth inhibition was calculated as the ratio between growth in the presence and absence of heavy metals, and compared to that observed for the wild-type strain under the same growth conditions.

### Swimming assay

Strains were grown overnight in LB medium, cells were concentrated to an OD_600_ = 3.0 and a 2-μl aliquot of the resulting cell suspension was laid onto the surface of soft M9 minimal medium agar plates [containing 0.3% (w/v) agar] added with glucose. Petri dishes were incubated at 30°C and the maximum diameter of the bacterial layer recorded after 48 h. A Δ*fliM*::mini-Tn*5* mutant (i.e., a flagellum-less strain) [81], was used as a negative swimming control.

### Biofilm formation tests

The gross ability of given strains for forming biofilms was examined with the crystal violet procedure described by O’Toole and Kolter [82]. Bacteria were grown at 30°C in 96-well plates (Nunclon™Δ Surface; Nunc A/S) on M9 minimal medium containing either glucose, citrate or glycerol. After 72 h, the wells were washed three times with water and treated for 15 min with 200 μl of 0.1% (w/v) crystal violet (Sigma-Aldrich Co.) in water. Wells were then washed twice with water, dried and the crystal violet bound to the surface-attached biomass eluted with 95% (v/v) ethanol. Biofilm formation index values were derived from *A*_595_ readings in a SpectraMax Plus384 Microplate Reader.

### UV sensitivity assay

UV sensitivities were determined in cells streaked out on square LB plates by means of a gradient of UV irradiation (254 nm, with exposure times varying from 0 to 100 s). Irradiations were performed with a germicidal lamp (model VL-6.MC; Vilber Lourmat, Marne-La-Vallée, France) at an intensity of 400 μW/cm^2^ (measured at 15 cm from the source).

### Resistance to toluene and heat shock assay

Fifty milliliters of LB medium was inoculated with 500 μl of an overnight culture of the corresponding strain, and cells were grown until an OD_600_ ~0.5 was reached. Fifty microliters of this culture was promptly taken, serially diluted 10-fold and plated onto LB agar plates. A 25-μl aliquot of toluene was immediately added to the remaining culture [resulting in a final toluene concentration of 0.05% (v/v) ≈ 5 mM] and the mixture was further incubated with agitation for 5 min. A similar procedure was followed to study resistance to heat shock. In this case, a mid-exponential culture obtained as detailed above was heated at 50°C for 5 min, and cells were immediately plated onto LB agar plates. In all cases, cells were plated onto LB agar plates, and colonies were counted after 24 h of incubation at 30°C. Survival rates were calculated from the number of colonies present before and after each treatment.

### Determination of cell viability by flow cytometry

Fluorescence-activated cell sorter cytometry analysis was performed to evaluate cell viability using PI. Flow cytometry analysis was performed in a Gallios^TM^ flow cytometer (Beckman Coulter Inc., Indianapolis, IN, USA), equipped with an argon ion laser of 15 mW at 488 nm as the excitation source. The PI fluorescence emission at 617 nm was detected using a 620/30-nm band pass filter array. PI (Life Technologies, Grand Island, NY, USA) was used from a stock solution at 0.5 mg/ml in water and added to a final concentration of 1.5 μg/ml to the cell suspension. Size-related forward scatter signals gathered by the cytometer were used by the Cyflogic^TM^ 1.2.1 software (CyFlo Ltd., Turku, Finland) to gate fluorescence data from bacteria in the stream. Ungated listmode data were collected and gating performed after the raw data files were collected. Data for 200,000 cells per experimental condition were collected, and the Cyflogic^TM^ 1.2.1 software was used to calculate the geometric mean of fluorescence per bacterial cell and the percentage of PI-positive cells in each sample. Viability was also side-tested by plating aliquots of the cell culture as explained in the preceding section.

### Determination of the mutation frequency

One hundred microliters of the 10^-5^, 10^-6^ and 10^-7^ dilutions of cells suspensions harvested 24 h after the culture reached their maximal OD_600_ values (to ensure that cells were well into the stationary phase) were seeded by quadruplicate onto LB agar plates, and 100 μl of each undiluted culture was spread by quadruplicate onto LB agar plates containing 250 μg/ml Rif. Plates were incubated at 30°C and colony counts were carried out after 48 h of incubation of the LB plates and after 36 h of incubation of the LB-Rif plates. Mutation frequency values are reported as the number of Rif-resistant colonies normalized to the total viable count for each condition.

### Construction of P_*rpoS*_ → *rpoS*‘-’*lacZ* translational fusion and measurement of β-galactosidase activity

The regulatory region of *rpoS* (774 bp upstream the first ATG) plus the sequence encoding the first 87 amino acids of the RpoS protein was amplified by PCR using oligonucleotides P_*rpoS*_-F and P_*rpoS*_-R (Table S1). The resulting 1,037-bp DNA fragment was digested with *Eco*RI and *Bam*HI and employed to replace the insert between the same sites in the broad-host-range plasmid pMCH1 [83]. This manipulation replaced the P_*fruB*_ promoter of pMCH1 with the P_*rpoS*_ promoter, giving rise to plasmid pMCH4, carrying the P_*rpoS*_ → *rpoS*‘-’*lacZ* translational fusion. Plasmid pMCH4 was then electroporated into *P*. *putida* KT2440 and its Δ*ppk* and Δ*ppx* derivatives, and analysed as explained below. The protocol described by Miller [36] was used to measure promoter activity. Cultures were developed as explained above (except that culture media were added with 150 μg/ml kanamycin to ensure plasmid maintenance), and the P_*rpoS*_ activity was determined during both exponential growth and in the stationary phase. A 1-ml aliquot of each culture was collected and promoter activity was measured by assaying the amount of β-galactosidase in cells made permeable with CHCl_3_ and sodium dodecyl sulfate.

### Statistical analysis

Reported experiments were independently repeated at least twice (as indicated in the corresponding figure legend), and the mean value of the corresponding parameter ± SD is presented. The statistical significance between multiple comparisons was obtained by the analysis of variance (ANOVA) followed by a Bonferroni post-test using, if necessary, transformed data. For the flow cytometry experiments, the median value is reported in box plots with the 1st and 3rd quartiles and the statistical significance was analyzed with the Mann–Whitney *U* test. In all cases, data were considered statistically significant when *P* < 0.05.

## Abbreviations

Pi: Phosphate; polyP: Polyphosphate; Ppk: Polyphosphate kinase; Ppx: Exopolyphosphatase; NMPs: Nucleoside monophosphates; NDPs: Nucleoside diphosphates; NTPs: Nucleoside triphosphates; ATP: Adenosine triphosphate; GTP: Guanoside triphosphate; UMP: Uridine monophosphate; AMP: Adenosine monophosphate; TMP: Thymidine monophosphate; ppGpp: Guanosine tetraphosphate; NADPH: Nicotinamide adenine dinucleotide phosphate, reduced form; OD600: Optical density measured at 600 nm; EDTA: Ethylenediaminetetraacetic acid; ORF: Open reading frame; UV: Ultraviolet; rpm: Revolutions per minute; PM: Phenotypic Microarray; PI: Propidium iodide; Rif: Rifampicin; SD: Standard deviation.

## Competing interests

The authors declare that they have no competing interests.

## Authors’ contributions

PIN and MC designed the study, carried out genetic and biochemical experiments and drafted the manuscript. EMG designed and constructed plasmids used in the complementation studies, and ACT helped with the construction of the mutant strains. VDL conceived the study, coordinated the work and wrote the manuscript. All authors read and approved the final manuscript.

## Supplementary Material

Additional file 1: Table S1 Oligonucleotides used in this work. **Figure S1:** Phenotypic changes detected by phenotypic microarray assays. Click here for file
