# Supplementary material for: Accumulation of inorganic polyphosphate enables stress endurance and catalytic vigour in Pseudomonas putida KT2440
Source: Microb Cell Fact. 2013 May 20;12:50. doi: 10.1186/1475-2859-12-50 (PMC3673903; doi:10.1186/1475-2859-12-50)
Supplement: Additional file 1: Table S1 — Oligonucleotides used in this work. Figure S1: Phenotypic changes detected by phenotypic microarray assays. [file 1475-2859-12-50-S1.pdf]

Table S1. Oligonucleotides used in this work.

Oligonucleotide[a]	Sequence (5'→3')	Restriction enzyme
Δ*ppx*-TS1-F	CGG AAT TCC AGT CGG GCG TGA AAA TCG ACT TGG	*Eco*RI
Δ*ppx*-TS1-R[b]	GTT AAC GAC CGA GAA CAA CCA GAT CTC AGC GTA CGT TGA GGA CCG GGT T	
Δ*ppx*-TS2-F	GAT CTG GTT GTT CTC GGT CGT TAA C	
Δ*ppx*-TS2-R	CGT CTA GAG GAA GCG ATG TCG TCC AGA ACC GGA	*Xba*I
Δ*ppk*-TS1-F	CGG AAT TCC CAG ATT GAT ATC CAT GTA ACC GCG	*Eco*RI
Δ*ppk*-TS1-R[b]	CGG CTT CGT CCT CAG CGT ACG TTG ACT CTT CTG GGA GCT CCT GAG CAT CC	
Δ*ppk*-TS2-F	TCA ACG TAC GCT GAG GAC GAA GCC G	
Δ*ppk*-TS2-R	CGT CTA GAG CAC GAA ATC GGG CTG GAC ATT GCC	*Xba*I
pSW-F	GGA CGC TTC GCT GAA AAC TA	
pSW-R	AAC GTC GTG ACT GGG AAA AC	
P_{rpoS}-F	CCG GGA ATT CGA CAC CCT GTT CTC CAT CG	*Eco*RI
P_{rpoS}-R	CCG CGG ATC CAA GCG CGC AAA ATG CAC TTC	*Bam*HI
ppk-F[c]	CGC GGG AAT TCA Gga aag gaT ATA **ATG** CTG CAG ACC CC	*Eco*RI
ppk-R	CGC GGG GAT CCT TAT CAG CGT ACG TTG AGG AC	*Bam*HI

[a] Recognition sites for the restriction enzymes indicated are underlined in the oligonucleotide sequence.
[b] Complementary sequences added to the oligonucleotides in order to generate DNA fragments for gene deletion by sewing (cross-over) PCR are shown in red.
[c] A Shine-Dalgarno motif (lowercase) was added to the sequence in the forward oligonucleotide used for *ppk* cloning, and the *ppk* start codon is highlighted in boldface.

Figure S1. Phenotypic changes detected by phenotypic microarray assays.

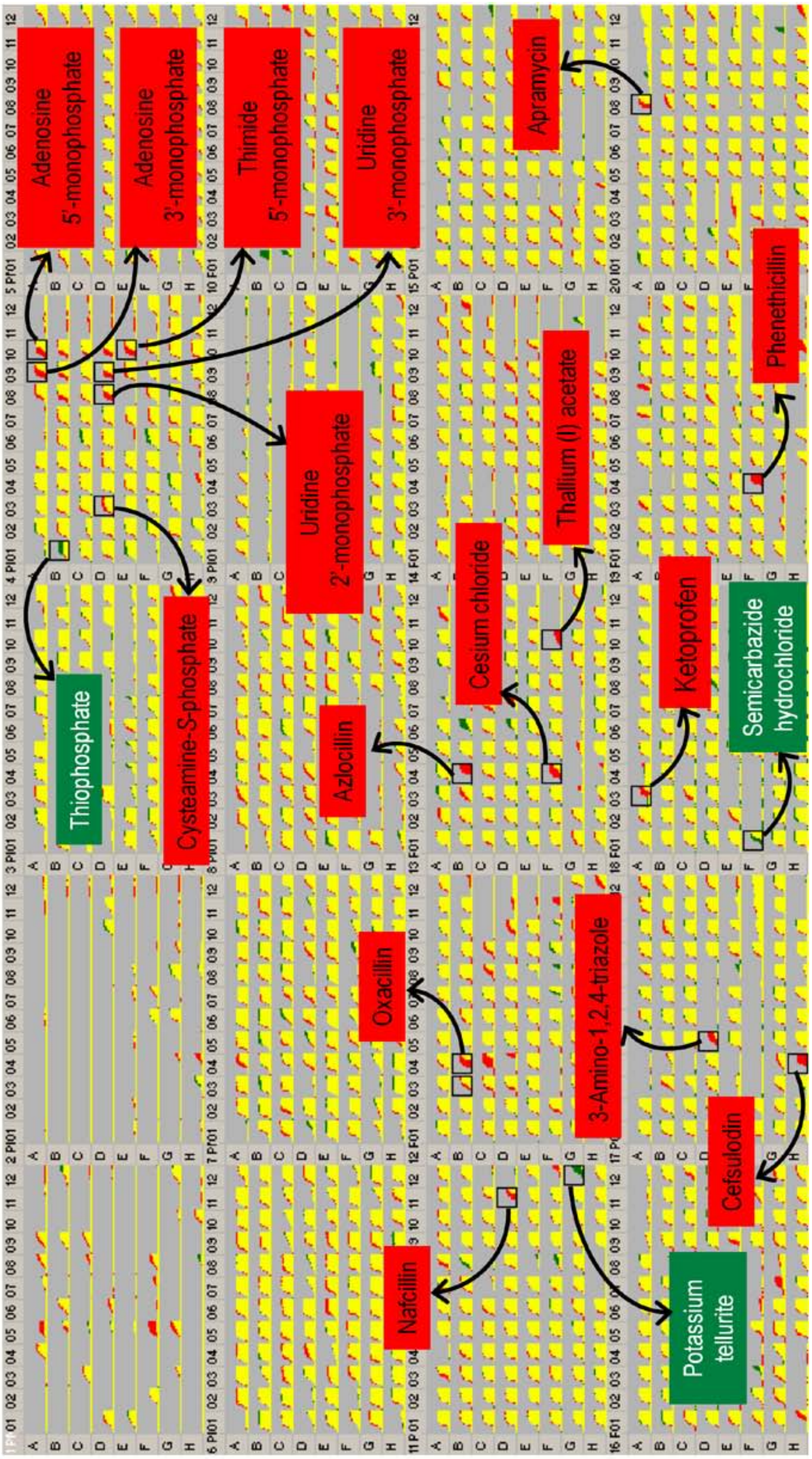

Significant changes (also shown in Table 1 in the main text) are enclosed in boxes and indicated by arrows. Yellow boxes indicate that respiration levels in the wild-type strain and that of the Δ*ppk* mutant was similar for the condition being testes. Red boxes indicate higher respiration rates of the wild-type strain than the mutant strain and, conversely, green boxes indicate higher respiration rates of the mutant strain as compared to the parental strain.
